# Rapid microbiological screening for tuberculosis in HIV-positive patients on the first day of acute hospital admission by systematic testing of urine samples using Xpert MTB/RIF: a prospective cohort in South Africa

**DOI:** 10.1186/s12916-015-0432-2

**Published:** 2015-08-14

**Authors:** Stephen D. Lawn, Andrew D. Kerkhoff, Rosie Burton, Charlotte Schutz, Gavin van Wyk, Monica Vogt, Pearl Pahlana, Mark P. Nicol, Graeme Meintjes

**Affiliations:** Department of Clinical Research, Faculty of Infectious and Tropical Diseases, London School of Hygiene & Tropical Medicine, Keppel Street, London, WC1E 7HT UK; The Desmond Tutu HIV Centre, Institute of Infectious Disease and Molecular Medicine, Faculty of Health Sciences, University of Cape Town, Cape Town, South Africa; Department of Medicine, Faculty of Health Sciences, University of Cape Town, Cape Town, South Africa; Department of Medicine, University of California San Francisco School of Medicine, San Francisco, CA USA; Department of Global Health, Academic Medical Center, Amsterdam Institute for Global Health and Development, University of Amsterdam, Amsterdam, The Netherlands; GF Jooste Hospital, Manenberg, Cape Town, South Africa; Khayelitsha District Hospital, Cape Town, South Africa; Clinical Infectious Diseases Research Initiative, Institute of Infectious Disease and Molecular Medicine, University of Cape Town, Cape Town, South Africa; Division of Medical Microbiology and Institute for Infectious Diseases and Molecular Medicine, Faculty of Health Sciences, University of Cape Town, Cape Town, South Africa; National Health Laboratory Service, Groote Schuur Hospital, Cape Town, South Africa; Department of Medicine, Imperial College, London, UK

**Keywords:** Tuberculosis, Pulmonary, Extrapulmonary, HIV, Diagnosis, Screening, Urine, Xpert, Africa

## Abstract

**Background:**

Autopsy studies of HIV/AIDS-related hospital deaths in sub-Saharan Africa reveal frequent failure of pre-mortem diagnosis of tuberculosis (TB), which is found in 34–64 % of adult cadavers. We determined the overall prevalence and predictors of TB among consecutive unselected HIV-positive adults requiring acute hospital admission and the comparative diagnostic yield obtained by screening urine and sputum samples obtained on day 1 of admission with Xpert MTB/RIF (Xpert).

**Methods:**

To determine overall TB prevalence accurately, comprehensive clinical sampling (sputum, urine, blood plus other relevant samples) was done and TB was defined by detection of *Mycobacterium tuberculosis* in any sample using Xpert and/or mycobacterial liquid culture. To evaluate a rapid screening strategy, we compared the diagnostic yield of Xpert testing sputum samples and urine samples obtained with assistance from a respiratory study nurse in the first 24 h of admission.

**Results:**

Unselected HIV-positive acute adult new medical admissions (n = 427) who were not receiving TB treatment were enrolled irrespective of clinical presentation or symptom profile. From 2,391 cultures and Xpert tests done (mean, 5.6 tests/patient) on 1,745 samples (mean, 4.1 samples/patient), TB was diagnosed in 139 patients (median CD4 cell count, 80 cells/μL). TB prevalence was very high (32.6 %; 95 % CI, 28.1–37.2 %; 139/427). However, patient symptoms and risk factors were poorly predictive for TB. Overall, ≥1 non-respiratory sample(s) tested positive in 115/139 (83 %) of all TB cases, including positive blood cultures in 41/139 (29.5 %) of TB cases. In the first 24 h of admission, sputum (spot and/or induced samples) and urine were obtainable from 37.0 % and 99.5 % of patients, respectively (*P* <0.001). From these, the proportions of total TB cases (n = 139) that were diagnosed by Xpert testing sputum, urine or both sputum and urine combined within the first 24 h were 39/139 (28.1 %), 89/139 (64.0 %) and 108/139 (77.7 %) cases, respectively (*P* <0.001).

**Conclusions:**

The very high prevalence of active TB and its non-specific presentation strongly suggest the need for routine microbiological screening for TB in all HIV-positive medical admissions in high-burden settings. The incremental diagnostic yield from Xpert testing urine was very high and this strategy might be used to rapidly screen new admissions, especially if sputum is difficult to obtain.

## Background

Tuberculosis (TB) is the leading cause of HIV/AIDS-related mortality [1], accounting for approximately 37 % of HIV/AIDS-related adult facility-based deaths in resource-limited settings [2]. Most of these deaths occur in sub-Saharan Africa where multiple hospital post-mortem studies conducted over the past 20 years have shown that between 34 % and 64 % of HIV-positive patients had TB at the time of death [2–7]. Disease at autopsy was predominantly disseminated and frequently remained undetected pre-mortem [2], reflecting failure of existing approaches to screening and TB diagnosis in HIV-infected medical in-patients.

The global TB control strategy defined in the mid-1990s was based on passive detection of sputum smear-positive pulmonary TB (PTB) in patients presenting with chronic cough [8]. However, in HIV-infected patients with TB and advanced immunodeficiency, chronic cough is less frequent, clinical presentation is non-specific, sputum smears are often negative and extrapulmonary TB (EPTB) is common [9, 10]. We therefore hypothesized that all HIV-positive patients requiring medical admission to hospitals in sub-Saharan Africa might warrant systematic routine microbiological investigation for TB regardless of clinical presentation. Second, we hypothesized that useful incremental diagnostic yield might be gained from additional testing of a non-respiratory sample such as urine, which is easier to obtain than sputum.

The advent of semi-automated rapid molecular tests provides new effective means of diagnosing HIV-associated TB. The Xpert MTB/RIF (Xpert) assay was endorsed by the World Health Organization (WHO) in 2010 for diagnosis of TB from sputum [11] and guidelines were updated in 2013 to include TB diagnosis from a range of non-respiratory samples, including cerebrospinal fluid and tissue needle aspirates/biopsies [12]. Endorsement was not given for testing other sample types due either to low sensitivity (pleural fluid) or for sample types for which there were insufficient data (stool, urine and blood). Importantly, however, assay specificity was found to be uniformly high regardless of sample type [12–14]. Moreover, two studies using Xpert to test urine samples from HIV-infected patients with advanced immunodeficiency each reported very promising results with high specificity (98 % and 100 %) and useful diagnostic yield, especially among in-patients [15, 16].

The present study conducted in a South African district hospital first aimed to accurately determine the prevalence and predictors of microbiologically confirmed TB among unselected HIV-positive medical admissions using microbiological screening. We then planned to use these data to define which patient sub-groups were at highest risk of TB and might warrant routine microbiological investigation at the time of hospital admission. Finally, with the ultimate aim of defining an effective rapid microbiological screening strategy on the first day of hospital admission among unselected HIV-positive admissions, we compared the diagnostic yield (proportions of total diagnoses) that could be made from rapid testing sputum and urine samples obtained in the 24-h period following admission using Xpert MTB/RIF.

## Methods

### Setting and patients

A prospective observational study was conducted at GF Jooste Hospital in Cape Town, South Africa. This 200-bed adult district hospital serves township communities of around 1.3 million people and with high HIV seroprevalence. This pre-defined study protocol was approved by the human research ethics committees of the University of Cape Town, South Africa, and the London School of Hygiene & Tropical Medicine, UK. Patients provided written informed consent in their first language.

Adult patients aged ≥18 years were recruited on 4 days of each week from both male and female medical wards. On recruitment days, the study coordinator ascertained from the ward register all medical admissions in a 24-h period and recorded these in the study register. All patients with previously negative or undocumented HIV status were offered testing using two rapid tests. All patients with a positive test (either a new or an existing test result) were eligible and invited to participate in the study. Patients receiving TB treatment at the time of hospital admission, however, were excluded.

### Procedures and samples

Demographic and clinical details (including the WHO symptom screen for HIV-associated TB [17]) were recorded as were medical history, use of antiretroviral therapy (ART), TB treatment and isoniazid preventive therapy (IPT). Patients were systematically investigated by the research team by obtaining sputum, urine and blood specimens for mycobacteriology between 9.00 am on the day of enrolment and 9.00 am the following day. Numerous additional samples for mycobacteriology were requested by the routine medical team as clinically indicated during the patient’s admission. These included sputum and a wide range of non-respiratory samples (Table 1).

**Table 1:** Clinical samples sent for mycobacteriology

Sample	Urine and sputum samples obtained in first 24 h		Total samples obtained during admission				
	Number of patients producing ≥1 sample (%)	Total number of samples	Number of patients producing ≥1 sample (%)	Total number of samples	Total number of culture and Xpert tests done	Number of positive culture and Xpert tests (%)	Number of TB patients with ≥1 positive culture or Xpert test (%)
Sputum	158 (37.0)	279	245 (57.4)	615	871	210 (24.1)	75 (54.0)^a^
Urine	418 (97.9)	418	418 (97.9)	418	833	141 (16.9)	89 (64.0)
Other non-respiratory samples	-	-	418 (97.9)	712	687	91 (13.2)	69 (49.6)
Ascitic fluid	-	-	5 (1.2)	5	5	1 (20.0)	1 (0.7)
Blood	-	-	410 (96.0)	469	469	41 (8.7)	41 (29.5)
Bone marrow	-	-	2 (0.5)	2	2	0	0
Cerebrospinal fluid (CSF)	-	-	76 (17.8)	94	94	8 (8.5)	8 (5.8)
Fine needle aspirate (FNA)	-	-	19 (4.4)	23	10	6 (60.0)	6 (4.3)
Gastric lavage	-	-	5 (1.2)	7	7	2 (28.6)	1 (0.7)
Pus	-	-	5 (1.2)	6	6	4 (66.7)	3 (2.2)
Pleural fluid	-	-	21 (4.9)	29	29	17 (58.6)	13 (9.4)
Stool^b^	-	-	9 (2.1)	10	0	0	0
Urine^c^	-	-	60 (14.1)	63	61	11 (18.0)	11 (7.9)
Other	-	-	4 (0.9)	4	4	1 (25.0)	1 (0.7)
Total	420 (98.4)	697	427 (100)	1,745	2,391	442 (18.5)	139 (100)

^a^Only 39 (28.1 %) of total diagnoses could be made from sputum samples obtained in the first 24 h; ^b^tested by Ziehl–Neelsen staining only; ^c^urine samples requested later than the first 24-h period for culture (not part of Xpert screening strategy)

In the first 24 h of admission, two sputum samples were requested from each patient with careful instruction and supervision by the study coordinator who was a trained respiratory nurse. A spot specimen was obtained first followed by a second sample that was induced using nebulized 3 % hypertonic saline. If necessary, both specimens were induced. Alternatively, for patients too unwell for sputum induction (for example, those with respiratory failure, bronchospasm or other danger signs), two spot specimens were requested instead. Urine samples were systematically collected using single-use disposable bed pans (Litha Healthcare Group, Johannesburg, South Africa) and a sterile syringe was used to transfer 50 ml to a polypropylene tube (Becton Dickinson, Sparks, MD, USA). Fresh aliquots (2.0 ml) were sent for immediate Xpert testing and the remaining urine was stored at −20°C for repeat Xpert testing after defrosting and concentration by centrifugation. Venous blood (5.0 ml) was inoculated into BACTEC™ Myco/F Lytic culture vials (Becton Dickinson, Franklin Lakes, NJ, USA).

### Laboratory procedures

Specimens were processed using standardized protocols and quality assurance procedures in centralized accredited laboratories of the South African National Health Laboratory Service (NHLS) as described elsewhere [18, 19]. Decontaminated, centrifuged deposits of sputum samples obtained in the first 24 h were resuspended in phosphate buffer and equal volumes were tested using culture and Xpert MTB/RIF. Mycobacterial growth indicator tubes (MGIT; Becton Dickinson) were inoculated and incubated for up to 6 weeks. Culture isolates were identified as *Mycobacterium tuberculosis* complex with the MTBDR*plus* line probe assay (Hain Lifescience, Nehren, Germany).

Additional sputum samples requested by the medical team were tested by MGIT culture and/or Xpert according to prevailing policy. Blood cultures from all patients were done in BACTEC™ Myco/F Lytic culture vials and other non-respiratory samples, such as pleural fluid, cerebrospinal fluid and tissue fine needle aspirates, were tested using MGIT culture.

Urine was tested using Xpert in two ways. Fresh urine samples (2.0 ml) were centrifuged and resuspended in 0.75 ml of phosphate buffer and then tested using the Xpert MTB/RIF assay as previously described [15]. In light of study-related logistical considerations and laboratory workflow, batches of frozen urine samples were defrosted and tested on a weekly basis. Each urine sample of between 30 ml and 40 ml was defrosted and centrifuged at 3,000 *g* for 15 min. Following removal of the supernatant, the pellet was resuspended in the residual urine volume and 0.75 ml was tested using Xpert.

### Data analysis

The proportions of patients able to produce urine and/or sputum samples during the first 24 h of admission were calculated and compared. New TB diagnoses were defined by detection of *M. tuberculosis* from any clinical sample obtained at any time during the admission period using MGIT culture or Xpert. The total yield of microbiologically confirmed TB diagnoses was used to calculate TB prevalence with 95 % exact confidence intervals (95 % CI). Then, using the total number of microbiological diagnoses as the denominator, we calculated the comparative yield of TB diagnoses from Xpert testing samples of urine and sputum obtained during the initial 24 h of admission. In addition, the proportions of patients whose TB diagnoses were derived from testing sputum samples (pulmonary TB (PTB)) and/or non-respiratory samples (extrapulmonary TB (EPTB)) were compared and these data were displayed using Venn diagrams.

Patients were characterized using simple descriptive statistics. Moderate and severe anaemia was defined using WHO criteria (haemoglobin ≤10.9 g/dL for both males and females) [20]. Medians were compared using either Wilcoxon rank-sum tests or Kruskal–Wallis tests as appropriate and means were compared using unpaired t-tests. Chi-squared, Fisher’s exact and McNemar’s tests were used as appropriate to compare proportions. Logistic regression analyses were used to identify patient factors associated with TB diagnosis. All variables in the univariable model meeting a cut-off of *P* ≤0.1 were included in the multivariable model. Statistical tests were two-sided at α = 0.05.

## Results

### Patients

**Fig. 1:**
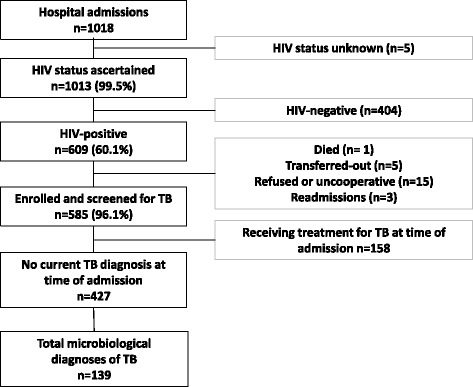
Flow diagram showing the study population

Between 6th June 2012 and 4th October 2013, the HIV status of 1,013 of 1,018 (99.5 %) unselected new admissions to the adult medical wards was ascertained (Fig. 1). Of the 609 patients who were HIV-positive, 585 (96.1 %) were enrolled in the study and investigated for TB. Those already receiving treatment for an existing diagnosis of TB at the time of admission (n = 158) were excluded, leaving 427 patients eligible for inclusion. These were typically young adults of whom a majority knew their positive HIV status before admission and more than one half had previously started ART (Table 2). Immunodeficiency was typically advanced and CD4 cell counts (median, 149 cells/μL) were lower among those who were ART-naive compared to those currently receiving ART (115 cells/μL versus 212 cells/μL; *P* <0.001). Two-thirds of all patients had moderate or severe anaemia. A history of previous TB was very common. No patients had received IPT. The vast majority of all patients had a positive WHO TB symptom screen regardless of TB status.

**Table 2:** Patient characteristics

Characteristic	All patients	Patients with a TB diagnosis	Patients without a TB diagnosis	*P* value
	(n = 427)	(n = 139)	(n = 288)	
Age (years), median (IQR)	36.1 (28.9–42.4)	33.8 (27.2–39.7)	37.1 (30.0–44.0)	0.008
Female	259 (60.7)	90 (64.8)	169 (58.7)	0.229
New HIV diagnosis on admission	82 (19.2)	34 (24.5)	48 (16.7)	0.055
History of previous TB^a^	196 (46.1)	50 (36.2)	146 (50.9)	0.005
ART status				
ART-naive	177 (41.5)	71 (51.1)	106 (36.8)	0.019
ART interrupted	71 (16.6)	19 (13.7)	52 (18.1)	
Current ART use	179 (41.9)	49 (35.3)	130 (45.1)	
If currently on ART, treatment duration (years), median (IQR)	1.6 (0.6–3.6)	1.3 (0.2–2.9)	1.7 (0.6–3.7)	0.157
CD4 cell count (cells/uL) (n = 424)				
Median (IQR) cells/μL	149 (55–312)	80 (33–182)	191 (73–392)	<0.001
<50	94 (22.2)	53 (38.4)	41 (14.3)	
50–99	66 (15.6)	21 (15.2)	45 (15.7)	
100–149	53 (12.5)	19 (13.8)	34 (11.9)	
150–199	42 (9.9)	16 (11.6)	26 (9.1)	
≥200	169 (39.9)	29 (21.0)	140 (49.0)	
Haemoglobin levels (n = 421)				
Median (IQR)	9.6 (7.7–11.6)	8.4 (6.8–10.2)	10.2 (8.2–12.2)	<0.001
No/mild anaemia	144 (34.2)	28 (20.1)	116 (41.1)	
Moderate/severe anaemia	277 (65.8)	111 (79.9)	166 (58.9)	
Symptoms				
Cough ≥2 weeks^a^	34 (8.0)	18 (13.0)	16 (5.6)	0.008
Sputum production^a^	161 (38.3)	51 (37.5)	110 (38.7)	0.808
Current cough^a^	201 (47.4)	84 (60.9)	117 (40.9)	<0.001
Current fever^a^	63 (14.9)	24 (17.5)	39 (13.6)	0.294
Current night sweats^a^	174 (41.0)	73 (52.9)	101 (35.3)	0.001
Current reported weight loss^a^	189 (44.5)	76 (55.1)	113 (39.4)	0.002
Positive WHO symptom screen^a^	389 (91.5)	134 (97.1)	255 (88.9)	0.005

Data presented are numbers (%) unless otherwise stated^. a^Between two and seven values missing. IQR, interquartile range

### Clinical samples obtained

During hospital admission, clinical samples for mycobacteriology were obtained from all study participants (n = 427) with a total of 1,745 samples (average, 4.1 samples per patient). At least two samples were obtained from all patients except one and these samples were from a median of three anatomic locations per patient (Table 1).

In the first 24 h of admission, urine samples were produced by 425 of 427 (99.5 %) patients, although seven samples were misplaced in transit to the laboratory, leaving 418 urine samples available for testing. In contrast, only 158 (37.0 %) patients were able to produce sputum in the same 24-h period (*P* <0.001) despite assistance from a respiratory nurse. Of these sputum samples, 37 (23.4 %) were obtained following sputum induction. The remaining patients who did not produce sputum were typically too sick or uncooperative to tolerate sputum induction, or it was unsuccessful.

Sputum was eventually produced by a total of 245 (57.4 %) patients at some point during their admission. The ability of patients to produce sputum samples was strongly related to the presence or absence of respiratory symptoms prior to admission. Of 161 patients who self-reported sputum production prior to admission, 143 (88.8 %) were able to produce a sputum sample for testing. Of 201 patients reporting current cough at admission, 159 (79.1 %) produced a sputum sample. Of 34 patients with chronic cough ≥2 weeks, 27 (79.4 %) produced a sputum sample.

A large number (n = 712) of various other non-respiratory samples were obtained (Table 1). A total of 2,391 Xpert and mycobacterial cultures were done on all the accumulated samples collected (mean, 5.6 tests per patient). The mean number of tests was similar for patients in whom TB was or was not diagnosed (5.8 tests versus 5.5 tests, respectively; *P* = 0.154). Results were missing for a small proportion of samples: 4.3 % (n = 12) of sputum cultures were contaminated; and 4.3 % (n = 12) of sputum Xpert tests and 3.1 % (n = 26) of urine Xpert tests gave indeterminate results.

### TB prevalence and risk factors

A microbiological diagnosis of TB was made in 139 patients (mean, 3.2 positive samples per case diagnosed) and the characteristics of those with and without TB are summarized in Table 2. The overall prevalence of TB was 32.6 % (95 % CI, 28.1–37.2; 139/427). Thus, the number of patients that had to be screened (number needed to screen (NNS)) to diagnose one additional case was just 3.1 (95 % CI, 2.7–3.6). Overall, ≥1 non-respiratory sample(s) tested positive (indicating extrapulmonary involvement) in 115/139 (83 %) of all TB cases, including positive blood cultures in 41/139 (29.5 %). When patients were stratified by various demographic or clinical characteristics or by presenting symptoms, TB prevalence ranged from 11.1 % to 52.9 %, and in univariable analyses was associated with younger adults, those with a new HIV diagnosis, those who were ART-naive, those with lower CD4 cell counts and those with moderate or severe anaemia (Table 3). The corresponding NNS in these groups ranged from 1.9 to 9.0.

**Table 3:** Tuberculosis (TB) prevalence stratified by patient characteristics and the number needed to screen (NNS) to identify one TB case in different risk groups

Characteristic	Number of TB cases	TB prevalence, % (95 % CI)	*P* value	NNS (95 % CI)
All patients	139	32.6 (28.1–37.2)	-	3.1 (2.7–3.6)
Age (years)				
<35 (n = 199)	75	37.7 (30.8–44.6)	0.034	2.7 (2.2–3.2)
≥35 (n = 228)	64	28.1 (22.3–34.4)		3.6 (2.9–4.5)
Gender				
Male (n = 168)	49	29.2 (22.4–36.6)	0.260	3.4 (2.7–4.5)
Female (n = 259)	90	34.8 (29.0–40.9)		2.9 (2.4–3.4)
HIV diagnosis				
New (n = 82)	34	41.5 (32.1–53.1)	0.055	2.4 (1.9–3.1)
Existing (n = 345)	105	30.4 (25.0–35.1)		3.3 (2.8–4.0)
Previous history of TB				
No (n = 229)	88	38.4 (32.1–45.1)	0.005	2.6 (2.2–3.1)
Yes (n = 196)	50	25.5 (19.6–32.2)		3.9 (3.1–5.1)
ART status				
Naive (n = 177)	71	40.1 (32.8–47.7)	0.007	2.5 (2.1–3.0)
Any ART exposure (n = 250)	68	27.2 (21.8–33.2)		3.7 (3.0–4.6)
CD4 cell count (cells/μL)				
<100 (n = 160)	74	46.3 (38.3–54.3)	<0.001	2.2 (1.8–2.6)
100–199 (n = 95)	35	36.8 (27.2–47.4)		2.7 (2.1–3.7)
≥200 (n = 169)	29	17.2 (11.8–23.7)		5.8 (4.2–8.5)
WHO anaemia grade				
No/mild anaemia (n = 144)	28	19.4 (13.3–26.9)	<0.001	5.2 (3.7–7.5)
Moderate/severe anaemia (n = 277)	111	40.0 (34.3–46.1)		2.5 (2.2–2.9)
Sputum production				
No (n = 259)	85	32.8 (27.1–38.9)	0.814	3.0 (2.6–3.7)
Yes (n = 161)	51	31.7 (24.6–39.5)		3.2 (2.5–4.1)
Current cough				
No (n = 223)	54	24.2 (18.7–30.4)	<0.001	4.1 (3.3–5.3)
Yes (n = 201)	84	41.8 (34.9–48.9)		2.4 (2.0–2.9)
Cough duration				
<2 weeks (n = 389)	120	30.8 (26.3–35.7)	0.008	3.2 (2.8–3.8)
≥2 weeks (n = 34)	18	52.9 (35.1–70.2)		1.9 (1.4–2.8)
Current fever				
No (n = 360)	113	31.4 (26.6–36.5)	0.294	3.2 (2.7–3.8)
Yes (n = 63)	24	38.1 (26.1–51.2)		2.6 (2.0–3.8)
Current night sweats				
No (n = 250)	65	26.0 (20.7–31.9)	0.001	3.8 (3.1–4.8)
Yes (n = 174)	73	42.0 (34.5–49.7)		2.4 (2.0–2.9)
Recent weight loss				
No (n = 236)	62	26.3 (20.8–32.4)	0.002	3.8 (3.1–4.8)
Yes (n = 189)	76	40.2 (33.2–47.6)		2.5 (2.1–3.0)
WHO symptom screen				
Negative (n = 36)	4	11.1 (3.1–26.1)	0.005	9.0 (3.8–32.3)
Positive (n = 389)	134	34.5 (29.7–39.4)		2.9 (2.5–3.4)

We next examined the predictive value of symptoms for identifying those with TB. Although the sensitivity of the WHO symptom screen [17] for HIV-associated TB was 97.1 % (92.7–99.2 %), the specificity was only 11.1 % (7.8–15.4 %) and the positive predictive value was just 34.5 % (29.7–39.4 %). In multivariable analyses, neither a positive WHO symptom screen nor a patient report of pre-admission sputum production was a significant independent predictor of TB diagnosis (Table 4). In contrast, lower CD4 cell counts and moderate and severe anaemia were independently associated with a higher risk of TB, while a history of previous treatment for TB was protective (Table 4).

**Table 4:** Multivariable logistic regression of patient characteristics associated with tuberculosis (TB) diagnosis

Characteristic	Unadjusted odds ratio	*P* value	Adjusted odds ratio	*P* value
Age (years)				
≥35	1	0.034	1	0.313
<35	1.55 (1.03–2.33)		1.26 (0.81–1.96)	
Gender				
Male	1	0.227	1	0.170
Female	1.29 (0.85–1.97)		1.40 (0.87–2.25)	
Previous history of TB				
Yes	1	0.004	1	0.038
No	1.82 (1.20–2.77)		1.66 (1.03–2.69)	
HIV diagnosis				
Existing	1	0.059	1	0.744
New	1.62 (0.99–2.66)		0.89 (0.46–1.75)	
ART status				
Ever received ART	1	0.005	1	0.206
Never received ART	1.79 (1.19–2.70)		1.44 (0.82–2.51)	
Sputum production				
Yes	1	0.808		
No	1.05 (0.69–1.61)			
WHO symptom screen				
Negative	1	0.002	1	0.068
Positive	4.20 (1.46–12.14)		2.59 (0.85–7.92)	
CD4 cell count (cells/μL)				
≥200	1	<0.001	1	<0.001
100–199	2.77 (1.56–4.93)		2.32 (1.26–4.26)	
<100	4.15 (2.50–6.89)		3.17 (1.81–5.56)	
Anaemia severity				
No/mild anaemia	1	<0.001	1	0.011
Moderate/severe anaemia	2.77 (1.72–4.47)		1.96 (1.16–3.36)	

### Diagnostic yield from urine and sputum samples obtained in first 24 h

**Fig. 2:**
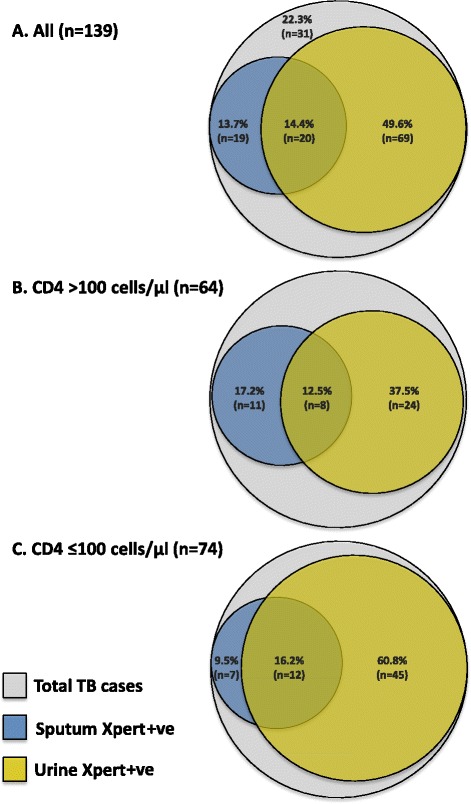
Tuberculosis (TB) diagnoses made by Xpert from urine and sputum samples collected in the first 24 h of hospital admission. Venn diagrams show diagnostic yields as proportions of (**a**) total TB diagnoses (n = 139), (**b**) TB diagnoses in patients with CD4 cell counts >100 cells/μL (n = 64) and (**c**) TB diagnoses in patients with CD4 cell counts ≤100 cells/μL (n = 74). Note: the CD4 cell count result was missing for one patient with TB

With the aim of defining a day 1 rapid microbiological screening strategy for new admissions, we next compared the diagnostic yields of Xpert when testing whatever sputum and urine samples could be obtained during the first 24 h of admission (Table 5). The number (%) of cases diagnosed from urine was substantially increased by prior concentration of urine by centrifugation (unconcentrated urine yielded 59 of 139 (42.4 %) diagnoses, whereas concentrated urine yielded 82 of 139 (59.0 %) diagnoses; *P* = 0.006). Overall, 64.0 % (89 of 139) of all cases could be diagnosed from urine. In contrast, only 39 (28.1 %) cases could be diagnosed by Xpert testing sputum (Fig. 2a) (*P* <0.001), reflecting in large part the small proportion of patients able to produce sputum in the first 24 h of admission. Combined urine and sputum Xpert results gave the greatest diagnostic yield, detecting 108 of 139 (77.7 %) cases (Table 5).

**Table 5:** Diagnostic yield of tuberculosis (TB) cases from different sample types and the proportion of diagnoses that could be made using Xpert testing of urine samples

Sample	Number of TB diagnoses	Diagnostic yield, % (95 % CI)	Number diagnosed by urine Xpert	Proportion diagnosed by urine Xpert^a^, % (95 % CI)
All clinical samples	139	100 (97.4–100)	89	64.0 (55.5–72.0)
Samples collected during the first 24 h of admission				
Urine				
Urine Xpert (unconcentrated)	59	42.4 (34.1–51.1)	59	-
Urine Xpert (concentrated)	82	59.0 (50.3–67.3)	82	-
Urine Xpert (either sample)	89	64.0 (55.5–72.0)	89	-
Sputum				
Xpert (first sample only)	37	26.6 (19.5–34.8)	20	54.1 (36.9–70.5)
Xpert (either sample)	39	28.1 (20.8–36.3)	20	51.3 (34.8–67.6)
Sputum and urine				
Xpert (all samples)	108	77.7 (69.8–84.3)	89	82.4 (73.9–89.1)
All other samples collected during admission				
Sputum				
Xpert	57	41.0 (32.7–49.7)	30	52.6 (39.0–66.0)
Culture	58	41.7 (33.4–50.4)	33	56.9 (43.2–69.8)
Either culture or Xpert	75	54.0 (453–62.4)	42	56.0 (44.1–67.5)
Cultures of non-respiratory samples				
All non-respiratory samples	70	50.3 (41.8–58.9)	46	65.7 (53.4–76.7)
Blood	41	29.5 (22.1–37.8)	32	78.0 (62.4–89.4)
Pleural fluid	13	9.4 (5.1–15.5)	6	46.2 (19.2–74.9)
Urine	11	7.9 (4.0–13.7)	8	72.7 (39.0–94.0)
Cerebrospinal fluid (CSF)	8	5.8 (2.5–11.0)	3	37.5 (8.5–75.5)
Lymph node fine needle aspirate (FNA)	6	4.3 (1.6–9.2)	4	66.7 (22.2–95.7)
Other	5	3.6 (1.2–8.2)	4	80.0 (28.4–99.5)

^a^Any positive result from testing unconcentrated and concentrated samples

The diagnostic yield obtained from urine but not sputum was associated with the CD4 cell count. In those with CD4 cell counts >100 and ≤100 cells/μL, the proportions diagnosed from sputum were similar (29.7 % versus 25.7 %, respectively; *P* = 0.70) whereas the yield from Xpert testing urine was substantially greater among those with lower CD4 cell counts (50.0 % versus 77.0 %, respectively; *P* = 0.001) (Fig. 2b,c).

### Diagnostic yield from total clinical samples collected during admission

**Fig. 3:**
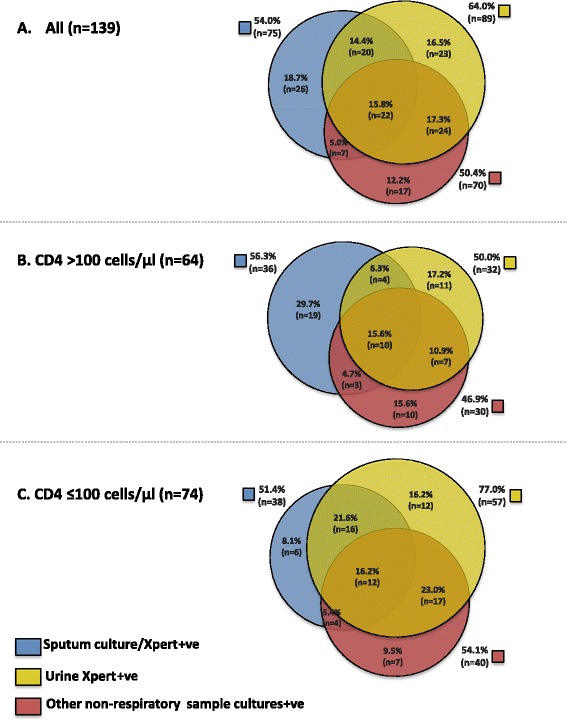
Yields of total tuberculosis (TB) diagnoses from all clinical samples collected at any time during hospital admission. Yields of TB diagnoses made by testing urine samples (using Xpert) collected on admission compared with the yield from all sputum samples (using either Xpert and/or culture) and all other non-respiratory samples (using culture). Venn diagrams show yields as proportions of (**a**) all TB diagnoses (n = 139), (**b**) TB diagnoses in patients with CD4 cell counts >100 cells/μL (n = 64) and (**c**) TB diagnoses in patients with CD4 cell counts ≤100 cells/μL (n = 74). Note: the CD4 cell count result was missing for one patient with TB

We next compared the diagnostic yield from Xpert tests done on admission urine samples (n = 418) with the yield from all other samples (615 sputum samples and 712 non-respiratory samples) collected during the entire period of hospital admission (Table 5 and Fig. 3). The yield from Xpert testing urine was slightly greater (but this did not reach statistical significance) than that obtained from the combined yield from Xpert and culture tests done on sputum samples (64.0 % versus 54.0 %, respectively; *P* = 0.146), but did exceed that from cultures of other non-respiratory samples (64.0 % vs 50.4 %, respectively; *P* = 0.028) (Fig. 3a).

Of 75 total microbiologically confirmed pulmonary TB (PTB) diagnoses, more than one half (n = 42; 56.0 %) could alternatively be diagnosed by testing urine with Xpert. Similarly, of the 70 microbiologically confirmed extrapulmonary TB (EPTB) diagnoses made using culture of non-respiratory samples, two-thirds (n = 46; 65.7 %) could be made by testing urine with Xpert. This proportion varied by sample type (Table 5). For example, whereas 78.0 % (32 of 41) of those with positive blood cultures tested urine Xpert positive, only 37.5 % (3 of 8) of those with culture-confirmed TB meningitis tested urine Xpert positive.

### Specificity of Xpert testing

We finally examined the possibility of false-positive sputum Xpert results in patients previously treated for TB disease who might potentially have had persistent shedding of non-viable organisms in sputum [21]. A positive Xpert test from one or more sputum samples was recorded in 57 patients with a TB diagnosis. Of these, 50 (88 %) had positive cultures from either sputum or a non-respiratory sample, thereby confirming a current diagnosis of active TB. Of the remaining seven patients, four had a history of previous TB treatment but only one of these had received treatment for TB within the preceding 3-year period.

## Discussion

Using comprehensive clinical sampling, a microbiological diagnosis of TB was made in one in three unselected HIV-positive new medical admissions to this South African district hospital. Since TB prevalence was so high and since neither clinical symptoms nor patient risk factors could be used to reliably predict who did and who did not have TB, we suggest that a policy of routine microbiological investigation of all HIV-positive medical admissions may be justified in this and other high-burden settings. Urine samples were readily obtained from patients in the first 24 h of admission and testing these with Xpert was the single investigation that yielded the greatest number of TB diagnoses, representing nearly two-thirds of total TB diagnoses. Use of Xpert to screen sputum samples collected in the first 24 h provided a substantially lower yield, reflecting the well-recognized difficulty of obtaining sputum from sick in-patients, especially when they have not been pre-selected based on respiratory symptoms. Routine testing of urine samples with Xpert provides an effective means of rapid TB diagnosis in this patient population and one that could readily be implemented wherever the GeneXpert test platform is already being used. Consideration should be given to routine testing of urine as the initial diagnostic screen in such patients, and especially in those for whom sputum production is difficult.

Patients were investigated very thoroughly for TB with an average of 5.6 tests done on samples obtained from a median of three anatomic compartments in each patient. Thus, this is likely to have provided a very reliable estimate of TB prevalence in these unselected patients. Positive non-respiratory samples indicated extrapulmonary involvement in a large majority (83 %) of TB cases. Indeed, disease was frequently disseminated as shown by positive blood cultures in 30 % of TB patients and the observation that samples from more than one anatomic site tested positive for TB in approximately half of all TB cases (Fig. 3).

The prevalence of TB varied by risk categories but in none of the stratified groups was the prevalence less than 10 % (Table 3). Admission symptoms were very poorly predictive of undiagnosed TB and were not significantly associated with TB in multivariable analysis. Thus, we propose that in such clinical populations, patients should be investigated for TB regardless of their symptoms. While multiple studies of unselected out-patients screened prior to starting ART in South Africa have reported TB prevalence rates of approximately 20–25 % [18, 22–24], comparable studies of unselected hospital in-patients are lacking. In Lusaka, Zambia, the prevalence of sputum culture-positive TB was 27 % among HIV-positive medical in-patients, but these patients were selected on the basis of ability to produce sputum [25]. In the absence of a microbiological diagnosis, some patients might have received empirical TB treatment. However, since presentation was so very non-specific, such a strategy would inevitably miss many cases and TB treatment would be unlikely to be started within the first 24 h of admission.

Only approximately one third of all patients could produce a sputum sample within 24 h of admission despite assistance from a dedicated study respiratory nurse and availability of sputum induction [26]. Just over one half could produce sputum at any time during admission and this was strongly related to the presence or absence of respiratory symptoms at the time of admission. Thus, the low yield from sputum was directly related to the fact that patients were investigated for TB without pre-selection according to respiratory symptomatology. However, in a previous study of unselected HIV-positive ambulatory out-patients in this setting, the same research nurse using the same protocol obtained sputum from 90 % of patients at a single clinic visit [18, 26]. This also illustrates the well-recognized difficulties in obtaining sputum from hospital in-patients who are often too sick, weak or unable to cooperate. Thus, the very high comparative yield of urine-based TB diagnostics in this study was in large part a direct result of the ease with which urine could readily be obtained.

Prior centrifugation of between 30 ml and 40 ml of urine substantially increased the yield of cases and this observation is explained by the fact that Xpert detects the DNA associated with whole *M. tuberculosis* bacilli, which sediment with centrifugation, rather than detecting free DNA [27, 28]. The detection of *M. tuberculosis* bacilli in urine is strongly suggestive of renal involvement with TB, most likely arising as a result of haematogenous disease dissemination. The yield from urine was substantially greater among those with lower CD4 cell counts (Fig. 2b,c), likely reflecting higher mycobacterial load and greater risk of disease dissemination and renal involvement. Our findings are entirely consistent with a study from the USA early in the HIV epidemic, which reported a very high yield (77 %) of diagnoses from culture of urine samples from HIV-infected patients with advanced immunodeficiency and extrapulmonary TB [29]. However, compared to culture, which may take several weeks, Xpert testing takes just 2 h. The Determine TB-LAM urine lateral-flow assay for lipoarabinomannan is an alternative urine-based rapid TB diagnostic that has shown promise [30]. Although the diagnostic yield is lower than that of urine Xpert [16], its potential use at the point-of-care and the lack of need for instrumentation or electricity supply and its low cost are all very considerable advantages [30].

Xpert testing of urine could be used as a rapid screening investigation for TB among HIV-infected adults admitted to medical wards in high-burden settings. The optimum diagnostic algorithm for a given setting will depend on the disease burden and the financial resources and infrastructure available. In South Africa, a single Xpert cartridge is allocated per patient under national implementation. The greatest overall diagnostic yield in this clinical population would be obtained from testing concentrated urine samples and, if used to routinely screen all HIV-infected patients, approximately one in five tests would be positive. To gain the increased yield derived from sample centrifugation, testing would have to be laboratory-based and with appropriate biosafety facilities. Use of urine as the initial diagnostic sample might reduce the need to obtain respiratory and other non-respiratory samples. The advantages of this approach would include reducing the generation of infectious bio-aerosols in the ward environment, decreasing the need for biohazardous sputum induction or the need for invasive procedures to sample extra-pulmonary disease sites and reducing requirements for culture. Importantly, urine-based TB diagnosis identifies patients with the highest mortality risk [15, 16, 31, 32]; expediting TB diagnosis and treatment in this group may potentially improve survival. The impact of this screening strategy on survival and clinical outcomes is being evaluated in a large randomized controlled trial in South Africa and Malawi (Rapid urine-based Screening for Tuberculosis to reduce AIDS-related Mortality in hospitalized Patients in Africa (STAMP) trial; ISRCTN71603869).

Strengths of the present study include the investigation of an unselected sample of HIV-infected patients regardless of respiratory symptoms or clinical suspicion of TB or ability to produce a sputum sample. Microbiological investigation for TB was extremely thorough, testing a very large number of samples using quality-assured laboratories. Xpert has been demonstrated to have very high specificity when testing both respiratory and non-respiratory samples [12–14]. However, we carefully assessed the possibility of false-positive Xpert tests occurring among patients previously treated for TB and shedding non-viable organisms in sputum as being very low in this patient population. We also used single-use sterile receptacles for urine collection to reduce any risk of cross-contamination of urine samples with either live or dead *M. tuberculosis* bacilli.

Weaknesses include the fact that the study was conducted at a single site and the extent to which these findings can be generalized to other settings remains to be assessed. However, the remarkable uniformity in the findings of autopsy studies of HIV-infected in-patients conducted in West, East and southern Africa [2–7] and in India [33] suggest that these findings are likely to be broadly relevant across high-burden settings. The results should not be generalized to HIV-infected medical out-patients in whom the diagnostic yield is known to be lower [15].

## Conclusion

In conclusion, this study has defined a means of rapid microbiological diagnosis of HIV-associated TB through systematic screening in newly admitted HIV-positive medical in-patients in high TB burden settings. This urine-based approach overcomes the frequent difficulties in obtaining sputum samples from such patients. In addition to sputum testing, routine testing of urine samples with Xpert should be considered and further evaluated either as a first-line screening investigation to enhance the yield and speed of TB diagnosis in this clinical population or as an adjunctive investigation in patients who are sputum-scarce.

## Abbreviations

ART: Antiretroviral therapy
CI: Confidence interval
EPTB: Extrapulmonary tuberculosis
IPT: Isoniazid preventive therapy
MGIT: Mycobacterial growth indicator tube
NHLS: National Health Laboratory Service
NNS: Number needed to screen
PTB: Pulmonary tuberculosis
TB: Tuberculosis
WHO: World Health Organization
Xpert: Xpert MTB/RIF
